# Development and Characterization of Biodegradable Polymer Filaments for Additive Manufacturing

**DOI:** 10.3390/polym17243328

**Published:** 2025-12-17

**Authors:** Tomáš Balint, Jozef Živčák, Radovan Hudák, Marek Schnitzer, Miroslav Kohan, Maria Danko, Richard Staško, Peter Szedlák, Marek Jałbrzykowski, Katarzyna Leszczyńska, Pavol Alexy, Ivana Bírová, Zuzana Vanovčanová, Martina Culenová

**Affiliations:** 1 Biomedical Engineering and Measurement Department, Faculty of Mechanical Engineering, Technical University of Košice, Letná 1/9, 042 00 Košice, Slovakia; jozef.zivcak@tuke.sk (J.Ž.); radovan.hudak@tuke.sk (R.H.); marek.schnitzer@tuke.sk (M.S.); maria.danko@tuke.sk (M.D.); richard.stasko@tuke.sk (R.S.); peterszedlak@ymail.com (P.S.); 2 Faculty of Mechanical Engineering, Bialystok University of Technology, ul. Wiejska 45 c, 15-351 Bialystok, Poland; m.jalbrzykowski@pb.edu.pl; 3 Department of Medical Microbiology and Nanobiomedical Engineering, Medical University of Bialystok, 15-089 Bialystok, Poland; katarzyna.leszczynska@gumed.edu.pl; 4 Institute of Natural and Synthetic Polymers, Faculty of Chemical and Food Technology, Slovak University of Technology, Radlinského 9, 812 37 Bratislava, Slovakia; pavol.alexy@stuba.sk (P.A.); ivana.birova@stuba.sk (I.B.); zuzana.vanovcanova@stuba.sk (Z.V.); 5 Institute of Medical Biology, Genetics and Clinical Genetics of the Faculty of Medicine, Comenius University in Bratislava, Sasinkova 4, 811 08 Bratislava, Slovakia; martina.culenova@fmed.uniba.sk

**Keywords:** PLA/PHB blend, scaffold fabrication, 3D printing, mechanical testing, SEM analysis

## Abstract

In this study, the authors focus on optimizing the processing parameters for the fabrication of biodegradable polymer filaments intended for subsequent 3D printing of biomedical structures and implants. Following extrusion and additive manufacturing, the produced materials underwent a comprehensive evaluation that included mechanical, microbiological, biofilm formation, and electron microscopy analyses. The complexity of these tests aimed to determine the potential of the developed materials for biomedical applications, particularly in the field of scaffold fabrication. At the initial stage, three types of filaments (technical designations 111, 145, and 146) were produced using Fused Filament Fabrication (FFF) technology. These filaments were based on a PLA/PHB matrix with varying types and concentrations of plasticizers. Standardized destructive tensile and compressive mechanical tests were conducted using an MTS Insight 1 kN testing system equipped with an Instron 2620-601 extensometer. Among the tested samples, the filament labeled 111, composed of PLA/PHB thermoplastic starch and a plasticizer, exhibited the most favorable mechanical performance, with a Young’s modulus of elasticity of 4.63 MPa for 100% infill. The filament labeled 146 had a Young’s modulus of elasticity of 4.53 MPa for 100% infill and the material labeled 145 had a Young’s modulus of elasticity of 1.45 MPa for 100% infill. Microbiological assessments were performed to evaluate the capacity of bacteria and fungi to colonize the material surfaces. During bacterial activity assessment, we observed biofilm formation on the examined sample surfaces of each material from the smooth and rough sides. The colony-forming units (CFUs) increased directly with the exposure time. For all samples from each material, the Log10 (CFU) value reached above 9.41 during 72 h of incubation for the activity of each type of bacteria (*Staphylococcus aureus*, *Pseudomonas aeruginosa*, *Candida albicans*). Scanning electron microscopy provided insight into the surface quality of the material and revealed its local quality and purity. Surface defects were eliminated by this method. Overall, the results indicate that the designed biodegradable filaments, especially formulation 111, have promising properties for the development of scaffolds intended for hard tissue replacement and could also be suitable for regenerative applications in the future after achieving the desired biological properties.

## 1. Introduction

In recent years, increasing scientific attention has been devoted to the development of novel biodegradable materials and the exploration of their potential applications in the biomedical field. Polymers have emerged as highly attractive candidates for biomedical use, particularly in hard tissue engineering, due to their ability to elicit a controlled and beneficial host tissue response. To address the inherent limitations associated with single polymer systems, the concepts of polymer blending and polymer composite fabrication have been introduced. By combining two or more polymers, the resulting hybrid materials can exhibit enhanced mechanical, structural, and biological properties. Such polymer-based implants are widely employed in various medical interventions, particularly in orthopedic applications such as bone and joint prostheses, as well as in other fields where lightweight, biocompatible, and degradable materials are required. The continuous advancement of social and economic systems has further stimulated extensive scientific and engineering research into polymer discovery, optimization, and practical implementation. Recent innovations also emphasize the importance of tailoring polymer degradation rates to match tissue regeneration processes, thereby improving long-term implant performance. Moreover, the integration of bioactive molecules into polymer matrices is increasingly being explored to enhance biocompatibility and therapeutic outcomes [1,2,3,4,5,6,7,8,9]. Polylactic acid (PLA) is one of the most studied biopolymers because it can be produced from nontoxic renewable raw materials. PLA is extensively utilized in biomedical applications owing to its intrinsic biocompatibility and controlled biodegradability. Recent investigations have examined the incorporation of nanomaterials, particularly few-layer graphene, into PLA matrices to improve their mechanical and thermal performance while preserving cytocompatibility, thereby expanding the material’s potential for advanced biomedical use [10]. PHB is a biodegradable, biocompatible, and nontoxic aliphatic polyester, characterized by a highly regular, linear polymer chain that enables crystallization and confers favorable mechanical and thermal properties. As a microbial storage polymer synthesized primarily by various Bacillus and Ralstonia species, PHB exhibits a degradation profile compatible with physiological environments and generates nontoxic products such as 3-hydroxybutyric acid. Despite its advantageous biocompatibility and environmental sustainability, its intrinsic brittleness and narrow processing window have stimulated extensive research into copolymerization (e.g., with PHV) and blending strategies to enhance ductility, thermal stability, and suitability for biomedical applications including tissue engineering scaffolds, controlled drug delivery systems, and temporary implant materials [11]. Starch is a naturally occurring polysaccharide-based biopolymer synthesized by plants as a primary energy reserve. It is predominantly composed of two macromolecular fractions, linear amylose and highly branched amylopectin, whose structural arrangement strongly influences its physicochemical behavior. Starch is characterized by several advantageous material properties, including low density and low production cost, as well as its inherent biodegradability and non-abrasive nature, which make it an attractive renewable resource for various industrial and environmental applications [12,13,14,15,16]. The fabrication of filaments derived from biodegradable polymers introduces new perspectives in material development and represents a significant convergence of medical science and engineering disciplines. Such filaments enable the creation of specific patients’ biomedical devices through additive manufacturing, while ensuring biocompatibility and controlled degradation within physiological environments. Moreover, the integration of advanced polymer processing techniques facilitates the optimization of mechanical, thermal, and structural properties required for clinical applications. This multidisciplinary approach ultimately accelerates innovation in regenerative medicine, implant design, and personalized therapeutic solutions [17,18,19]. Using a desktop extrusion system, we produced three filaments with the type labels 111, 145, and 146 from the above-mentioned biodegradable components. Extrusion is a manufacturing process in which thermoplastic materials are forced through a die with a defined cross-sectional profile to produce a continuous strand of molded product (filament). The extrusion process begins with the material in the form of granules, pellets, or powders being fed from a hopper into the extruder zone. Then the melting process begins by means of the heat from mechanical energy generated by the rotation of the screw and the heaters that are placed along the head. The molten materials are then forced into the die, which structures the materials during the cooling process. Large companies use industrial filament makers (extrusion systems), which are characterized by large volumes of extruded material and precision. In our research, however, we chose desktop extrusion systems at a good level because desktop extrusion systems are increasingly on par with industrial extrusion systems in quality. When filament is used in additive technologies, it is essential that it meets the requirements needed for high-quality 3D printing of implants suitable for tissue substitution. Essential filament characteristics for additive manufacturing include high mechanical strength, uniform diameter consistency, and a smooth surface finish. These properties are critical determinants of the dimensional accuracy, structural integrity, and overall quality of the final printed constructs. Notably, the Fused Deposition Modeling (FDM/FFF) technique, which underpins much of modern polymer-based 3D printing, was first patented in 1989, providing the foundation for subsequent advancements in additive manufacturing technologies [20]. Three-dimensional printers based on FFF technology are currently the most popular three-dimensional printers. This technology is based on the extrusion of filament from a nozzle that is deposited on a heated plate, creating a two-dimensional layer on top of a second layer, resulting in a tangible three-dimensional object. FFF commonly employs filaments composed of thermoplastic polymers such as PLA, among others, with a prevalent filament diameter of 1.75 mm. PLA/PHB-based materials with plasticizer and starch are used as hard tissue substitutes, mainly because their properties can be well adapted to those of natural tissue and are biologically safe. The main reasons are biocompatibility and biodegradability, because both polymers gradually decompose into natural metabolites (lactic acid, 3-hydroxybutanoic acid), which is advantageous when the material is intended to replace newly formed bone mass. The PLA/PHB + starch and plasticizer composite allows the formation of a porous structure similar to trabecular bone. This supports the ingrowth of bone cells, the exchange of nutrients, and the formation of new bone mass [21,22]. These material characteristics facilitate the manufacture of functional implants via FFF, while maintaining biocompatibility and controlled resorption [23]. It is very difficult to produce a suitable medical filament that meets all these requirements. However, this study provides new findings and makes a significant contribution to the solution to this issue, as we subjected our materials to rigorous analysis using light microscopy techniques and an Olympus GX71 instrument (microscope) with an Olympus DP12 camera. We chose the SEM (secondary electron mode) and BSE (backscattered electron mode) imaging modes. Bioimplants designed for sites in the human body with low mechanical stress in the form of bioresorbable stents represent unique solutions in this field and bring new possibilities to prevent various complications. Research and development efforts are directed towards the use of biocompatible implants made of materials such as PLLA, PGA, PLGA, and PCL because of their mechanical strength and biocompatibility [24,25,26,27,28,29,30]. A key factor for long-term and reliable implant performance is good choice of biomaterial. Biological environments will not accept any material. Therefore, in order to optimize biological efficacy, implants should be chosen to reduce negative biological responses, while maintaining adequate function. To ensure the therapeutic efficacy of the implant, control of parameters is essential and three main factors should be considered: structure, mechanical properties, and biological properties [31]. Optimization of mechanical properties is very important for the in vivo functioning of the implant. With this in mind, manufacturing parameters must be optimized to ensure maximum mechanical functionality. The basic mechanical requirements of implants include elastic modulus and tensile, compressive, and shear strength, yield strength, fatigue strength, hardness, and toughness [32]. Mechanical characterization using an MTS Insight testing system, combined with microbiological assessments against various bacterial and fungal strains, produced statistically significant data that are synthesized in the subsequent sections. These dual modalities of testing provide a comprehensive evaluation of both the structural robustness of the samples and their resistance to microbial colonization under relevant biological conditions [33,34,35]. Based on the studies conducted, the researchers focused on the following composition/purpose: PLA/PHB/TPS of various formulations, intended for 3D-printable scaffolding applications. Mechanics (E, σu): the authors measured mechanical properties for various blends, where the results show that the mechanical values are within the usable range for scaffolds, with the addition of TPS typically reducing stiffness and tensile strength compared to pure PLA components. Dimensional tolerances/accuracy: the authors document printed scaffolds with well-preserved geometry and porosity. Printability (deformation/threading): PLA/PHB/TPS blends were considered printable (the author provides printing parameters—temperatures, speed) and the printing did not show significant clogging; at higher TPS content, layer bonding may deteriorate. Degradation: samples were tested in water/hydrolytically and the authors describe that TPS increases hydrophilicity and water absorption, potentially accelerating degradation; specific degradation intervals in biological environments are not fully quantified. Biological testing: MTT tests and direct contact cytotoxicity tests were performed on human chondrocytes without toxic effects; cells adhered and proliferated on the scaffold [36,37,38]. These studies show that PLA/PHB-based composites (with TPS and plasticizers or with bioactive fillers) are FDM-printable, exhibit mechanical properties suitable for 3D scaffolds, maintain the designed geometry and porosity, are biocompatible (no cytotoxicity, with cell adhesion and proliferation), and have potential as biodegradable tissue substitutes.

## 2. Materials and Methods

### 2.1. Preparation of PLA/PHB Filaments

**Figure 1:**
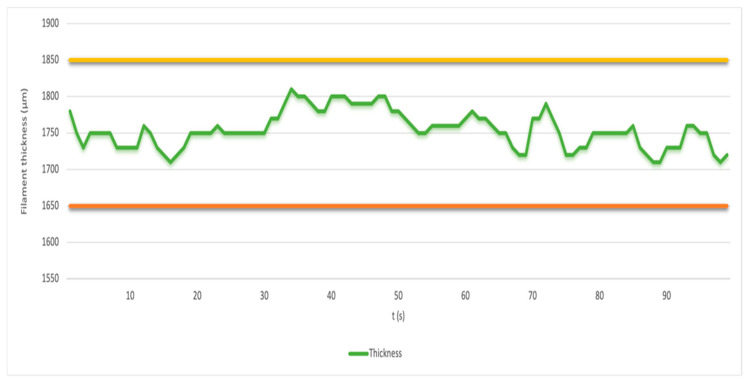
Graph of filament diameter with 25% plasticizer amount over time (t) where the yellow and red lines in the graph show the upper and lower permissible values.

The material for the production of biomedical filaments was delivered to the Department of Biomedical Engineering in the form of granules, vacuum packed in opaque packaging. However, we still carried out drying of the material in a dryer by 3devo (Utrecht, Netherlands). The drying temperature was set at 60 °C for 80 min. The actual filament production process was carried out on the 3devo device. The filament maker Composer 450 is a filament-making machine on which multiple materials can be mixed. The production took place in an air-conditioned room at 18 °C. Using HDPE (3devo) transit material, we cleaned the equipment before extrusion. The actual equipment cleaning process took several minutes. We then poured the granulate into the extruder tray. We set the melting temperature in the range of 160 °C to 180 °C for the production of the first medical filament with 25% plasticizer. For the production of the second filament with 30% plasticizer, we set the extrusion temperature on the heating elements to values ranging from 150 to 170 °C. For the last extrusion of the filament with the technical designation 145, we set the extrusion temperature range to 150 °C to 175 °C at 80% fan power. We achieved these temperatures by combining our knowledge of the melting temperature of PLA/PHB materials. After the filament flow had stabilized, we placed the extruded filament in a sensor that measured the filament diameter. By monitoring and optimizing all production parameters, the filament was then wound onto the spool. The filament production parameters are summarized in Table 1, Table 2 and Table 3. We were able to maintain the optimum filament diameter, as can be seen in Figure 1.

### 2.2. Preparation of Samples

**Figure 2:**
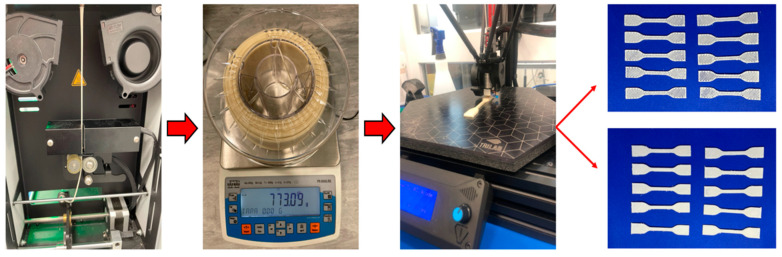
Process for obtaining samples for mechanical testing.

In this study, we printed samples using additive FFF technology (Figure 2). We optimized all the 3D printing parameters for the best results. The printing temperature was set to a value in the range of 160–175 °C and the plate temperature was set to a temperature range of 50–60 °C. FFF Printing Parameter Specification: layer height: 0.20 mm; fill pattern: 45-degree angle; infill percentages: 50%, 75%, and 100%; number of shells/walls: 3 circumferential shells (total wall thickness 1.2 mm); cooling settings: cooling fan activated at 100% after the first 3 layers; assembly orientation: samples printed in flat orientation (long axis parallel to the build plate); screen angle: ±45° relative to the thrust axis (bidirectional). The modeling and testing were carried out according to the relevant standard, EN ISO 527–2:1996 [39]. The ability of microorganisms to proliferate and form biofilms was observed on flat samples, each with a surface area of 4 cm^2^ and a thickness of 0.3 cm. All samples were modeled in SolidWorks 2024 (SP 5) software. The samples were printed from filaments composed of PLA/PHB/thermoplastic starch with the addition of type I and type II plasticizer, the type designations of which are shown in Table 4.

### 2.3. Physical Characterization

Mechanical tests are used to determine the properties that express the deformation behavior of the material and the failure conditions of the sample when subjected to external forces. Two types of samples were printed to perform mechanical testing. For the tensile test, this was the so-called dogbone type 5; a simple roller was printed for the compression test. The samples were printed with 100%, 75%, and 50% infill of materials with technical designations 111, 145, and 146, respectively. The tensile test was carried out on an MTS Insight 1 kN machine, using Instron 2620-601 (Norwood, MA, USA), with a 20 mm base and a feed rate of 2 mm/min. MTS Insight test equipment integrates electromechanical load frame technology in combination with TestWorks (4.6) software. The electromechanical drive system works with the aid of a control unit that provides highly accurate and repeatable results. The frame has incorporated load sensors that follow the IEEE 1451.4 standard [40]. The geometric specifications of the test specimens and the methodology for tensile testing were determined in accordance with the EN ISO 527-2:1996 [39]. This international standard establishes the parameters for evaluating the tensile properties of injection-molded and extruded polymeric materials, ensuring consistency and reproducibility of mechanical performance assessments. Compliance with this standard guarantees precise control over specimen dimensions, loading rates, and testing conditions, thereby facilitating reliable comparison of material behavior [41]. MTS Insight 1 kN and Instron 2620-601 equipment with a 12.5 mm base were used for the compression tests in the case of the tests with an extensometer. In the case of testing up to compression of the sample, MTS 858 Mini Bionix (Warren, MI, USA) was used because the force value exceeds the limit of the Insight machine. In both cases, the feed rate was 0.05 mm/s. From the mechanical tensile test, Young’s modulus of elasticity was determined, and this was based on Hooke’s law. The values were calculated from the linear load area for all infills used, which is approximately within 1/4 of the maximum load force. Cylindrical specimens with a diameter of 12 mm and a height of 18 mm (height to diameter ratio H/D ≈ 1.5) were selected to meet the requirements of ISO 604:2002 [42] and minimize the risk of buckling while remaining compatible with the accuracy of FDM printing and the limitations of layer adhesion. The chosen geometry ensures uniform load distribution, partially eliminates edge effects, and provides a sufficient cross-sectional area to capture representative material behavior. A minimum of *n* = 5 specimens was tested for each condition.

### 2.4. Microbiological Testing Characterization

The testing consists of assessing the ability of microorganisms to proliferate in the samples, each with an area of 4 cm^2^ and a thickness of 0.3 cm, marked Sample No. 111, Sample No. 145, and Sample No. 146. *Staphylococcus aureus*, *Pseudomonas aeruginosa*, and *Candida albicans-fungus*, also isolated from the environment, were used for the study. Bacterial and fungal strains were stored according to international standards using Cryobank (Mast Diagnostica, Mast Group Ltd., Bootle, UK). Prior to the experiment, the strains were activated in TSB nutrient liquid medium, and after 24 h incubation in an incubator at 35 °C under aerobic conditions, the strains were prepared into suspensions with appropriate inoculum. To assess the ability of the bacteria to proliferate in the presence of the test samples, a suspension with an inoculum of 105 bacterial cells/1 mL of Mueller–Hinton liquid medium was prepared from a 24 h culture of the reference bacteria. Before microbial testing, all samples were sterilized by UV irradiation and then hydrated in sterile saline. The cultures were incubated in an incubator at 36 ± 1 °C. After 24, 48, and 72 h of incubation, the number of colonies was verified by plating the suspensions quantitatively on Chapman agar (*S. aureus*) and cetrimide (*P. aeruginosa*). The mean bacterial count was then calculated for each sample. The test was performed in triplicate for each test material. Evaluation checks were introduced: bacterial growth in the medium used, sterility of the materials tested, and sterility of the medium used. To assess the ability of the fungi to proliferate in the presence of the test samples, suspensions with an inoculum of 104 cells/1 mL of Saboraud’s liquid medium were made from a 24 h culture of *C. albicans*. Test samples prepared as set out above were immersed in 3 mL of suspension transferred into sterile Petri dishes. The discrepancy between 20 mL for bacteria and 3 mL for fungi reflects the differences in microbial density, growth characteristics, and typical inoculation protocols for the two organisms. The cultures were incubated at room temperature at 21 ± 2 °C. After 24, 48, and 72 h of incubation, the number of colonies was verified by plating the suspensions quantitatively on Saboraud agar medium, and the mean number of surviving fungi was calculated for each sample. The test was carried out in triplicate for each test material. The evaluation checks were set as follows: fungal growth in the medium used, sterility of the materials tested, and sterility of the medium used.

### 2.5. Biofilm Testing Characterization

The test was carried out in sterile containers into which the prepared test samples were placed. Then, 20 mL of a suspension of *S. aureus*, *P. aeruginosa*, and *C. albicans* with an inoculum of 105 bacterial cells/1 mL of liquid medium and 200 μL of 1% TTC (2, 3, 5-triphenyltetrazolium chloride, SIGMA) solution was transferred into each container to evaluate the presence of red formazan produced by the reduction of TTC by metabolically active microorganisms [43]. This is a method that allows us to observe the process of biofilm formation by microorganisms. The sample containers were incubated in an incubation oven at 36 ± 1 °C under aerobic conditions for 7 days. After incubation, the samples were removed from the containers to remove the microorganisms forming the plankton suspension from their surfaces, the test samples were rinsed 3 times in sterile PBS at pH 7.2, and finally the samples were immersed in sterile deionized water. The test was performed in triplicate. Checks were set up to assess the sterility of the test materials and culture medium.

### 2.6. Electron Microscopy SEM/BSE Characterization

Using light microscopy techniques and Olympus GX71 (microscope) with an Olympus DP12 camera (Olympus, Tokyo, Japan), we tested the samples in the form of pellets which were made of filaments. The samples were cleaned using ultrasonication in methanol. We selected the SEM (secondary electron mode) and BSE (backscattered electron mode) imaging modes. A layer of gold was deposited on all analyzed samples before observation in the electron microscope. Pre-treatment of the samples consisted of preparation in dentacryl, grinding with sandpaper grits from 240 to 800, and polishing with diamond paste grits 1/0 and 1/4. Dentacryl dental acrylic resin was chosen for its rapid curing, ease of handling, and adequate mechanical support, which ensured proper stabilization of the scaffolds without affecting imaging quality. Subsequently, the samples were washed and rinsed with benzyl alcohol. In our study, the aim of SEM imaging was exclusively to evaluate the morphological features, surface topography, and microstructural homogeneity of PLA/PHB/TPS scaffolds.

## 3. Results and Discussion

### 3.1. Mechanical Characterization of Tensile Testing

**Figure 3:**
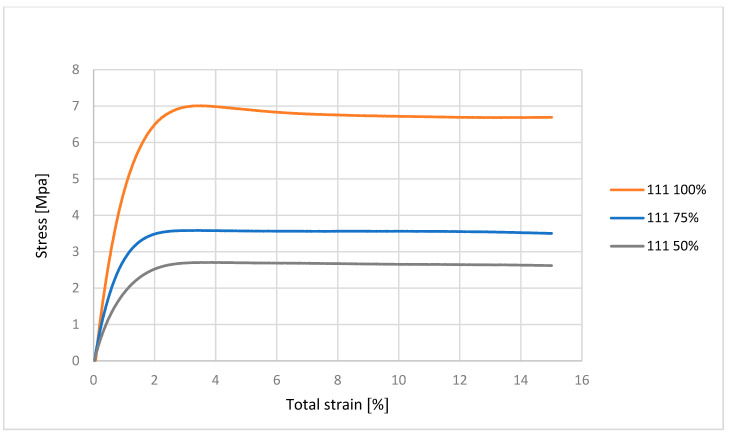
Graphical representation of the tensile test progress for material 111.

From Figure 3, we can evaluate that the material with 100% infill was up to approximately 5 MPa in the elastic deformation region, which represents approximately 1% of the relative elongation of the material. This means that if we remove the load, the material returns back to its original state, without permanent elongation. Permanent deformations only occur after the elastic limit has been exceeded. The material with 75% infill was within the elastic deformation range up to approximately 2 MPa. The material with 50% infill was in the region of elastic deformation up to approximately 1 MPa. The figure shows that the ability of the material to resist tensile forces is proportional to the decreasing cross-section of the material, i.e., the infill.

**Figure 4:**
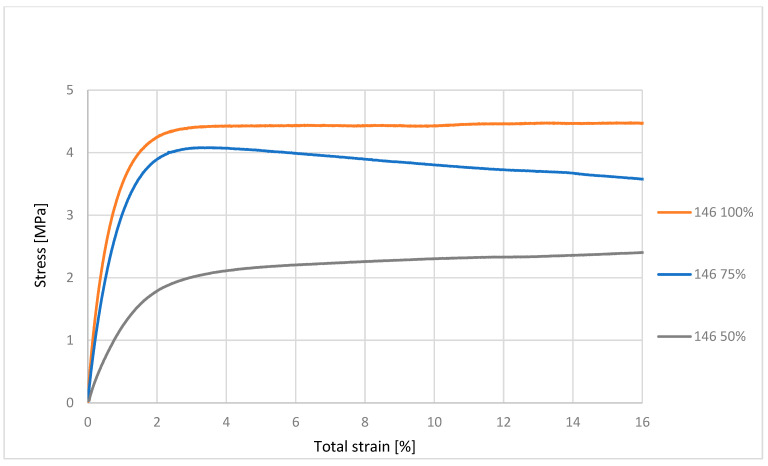
Graphical representation of the tensile test progress for material 146.

From Figure 4, we can evaluate that the material with 100% infill was up to approximately 3 MPa in the elastic deformation region, which is approximately 1% of the relative elongation of the material. This means that if we remove the load, the material returns to its original state without permanent elongation. Permanent deformations only occur after the elastic limit has been exceeded. The material with 75% infill was in the elastic deformation range up to approximately 2.5 MPa. The material with 50% infill was in the region of elastic deformation up to approximately 1 MPa. The figure shows that the ability of the material to resist tensile forces is proportional to the decreasing cross-section of the material, i.e., the infill.

**Figure 5:**
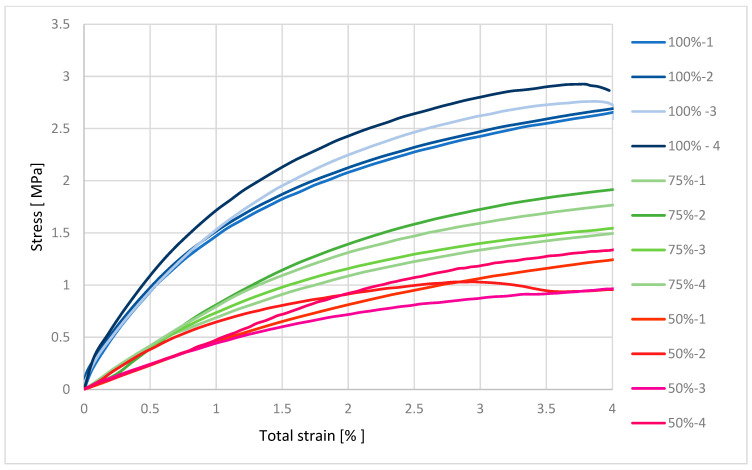
Graphical representation of the tensile test progress for material 145.

Figure 5 shows us that plastic deformation occurs earlier for the samples of material 145 than in the previous cases. The samples with 100% infill were in the region of elastic deformation up to approximately 1 MPa. For the samples with 75% and 50% infill, the elastic deformation region is minimal; the curve smoothly transitions to plastic deformation.

Based on Hooke’s law, Young’s modulus of elasticity values (Table 5, Table 6 and Table 7) were determined from the mechanical tensile test. The values were calculated from the linear load area for all the infills used, which is approximately at 1/4 of the maximum loading force. A comparison of the tested material with other materials, including biological ones, can be seen in Table 8 where the values are given in (MPa).

Table 8 above shows the values of Young’s modulus of elasticity in (MPa). Dental enamel has the highest value of 20 to 84 MPa. On the other hand, the lowest values of Young’s modulus of elasticity can be observed for healthy soft tissue, between 0.0005 and 0.007 MPa. Relevant values are achieved for the material with technical designation 111, the composition of which is PLA/PHB thermoplastic starch and a plasticizer. The value of Young’s modulus at 100% infill is around 4.63 MPa, it is 2.84 MPa at 75% infill, and the value is the lowest at 50% infill, i.e., 1.71 MPa. Also, in this research, we have shown that the value of Young’s modulus of elasticity decreases with decreasing infill. The values given show us that our material is suitable for soft tissue replacement.

### 3.2. Mechanical Characterization of Pressure Testing

**Figure 6:**
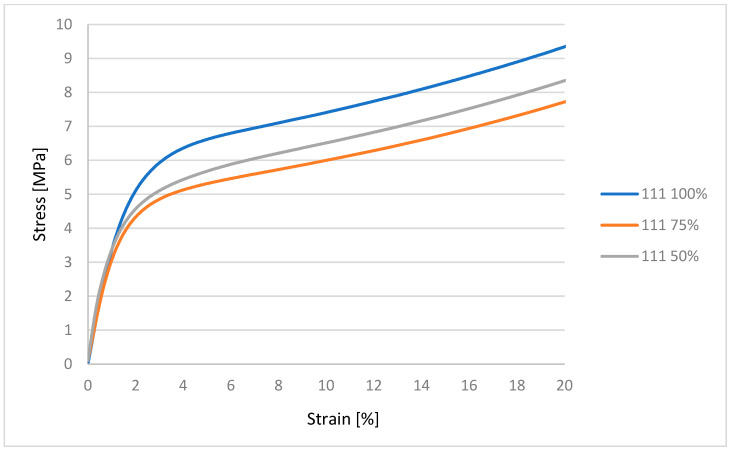
Graphical representation of the compression test for material 111.

It can be concluded from Figure 6 that the linear region for each porosity value is approximately the same. The material with 100% infill shows the highest resistance to compressive force. The region of plastic deformation occurs approximately up to the application of 4 MPa stress. The next regions of the graph indicate the region of plastic deformation, where progressive compression occurs without breaking the material. The graph shows that the ability of the material to resist compressive forces is proportional to the decreasing cross-section of the material, i.e., the infill.

**Figure 7:**
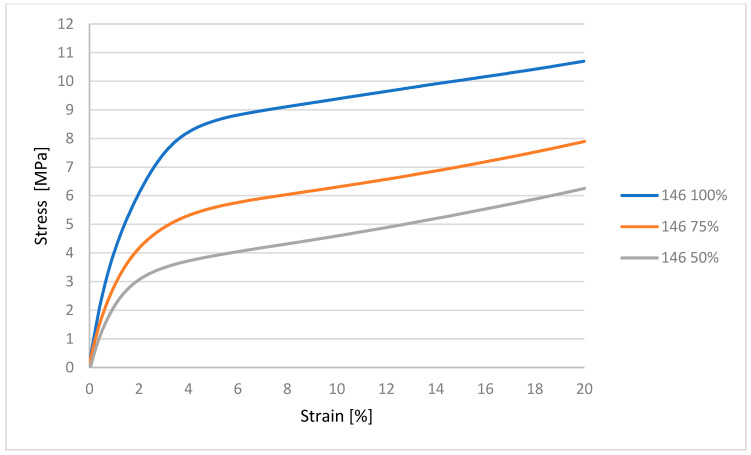
Graphical representation of the compression test for material 146.

It can be concluded from Figure 7 that the material with 100% infill shows the highest resistance to compressive force. The region of plastic deformation occurs approximately up to the application of 6 MPa stress. The next regions of the graph indicate the region of plastic deformation, where progressive compression occurs without breaking the material. The graph shows that the ability of the material to resist tensile forces is proportional to the decreasing cross-section of the material, i.e., the infill.

**Figure 8:**
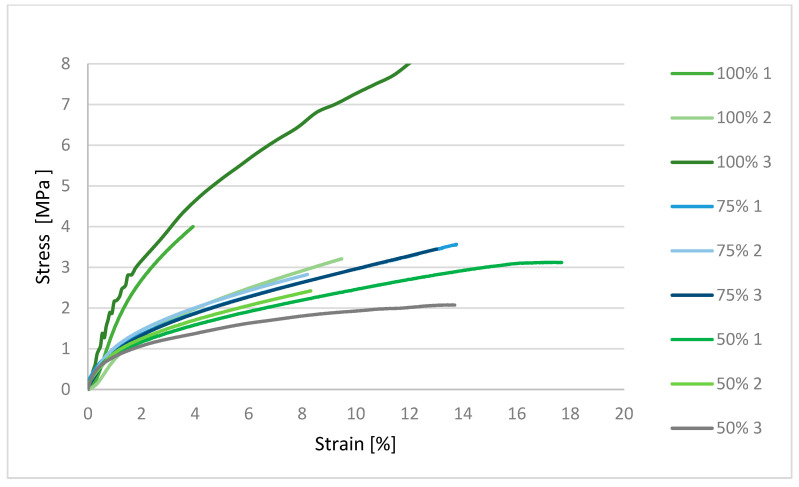
Graphical representation of the compression test for material 145.

Figure 8 shows that the material samples have low compressive resistance and smoothly transition into the deformation region. The material with 100% infill shows the highest resistance to compressive force. The graph shows that the ability of the material to resist tensile forces is proportional to the decreasing cross-section of the material, i.e., infill.

### 3.3. Microbiological Analysis

**Figure 9:**
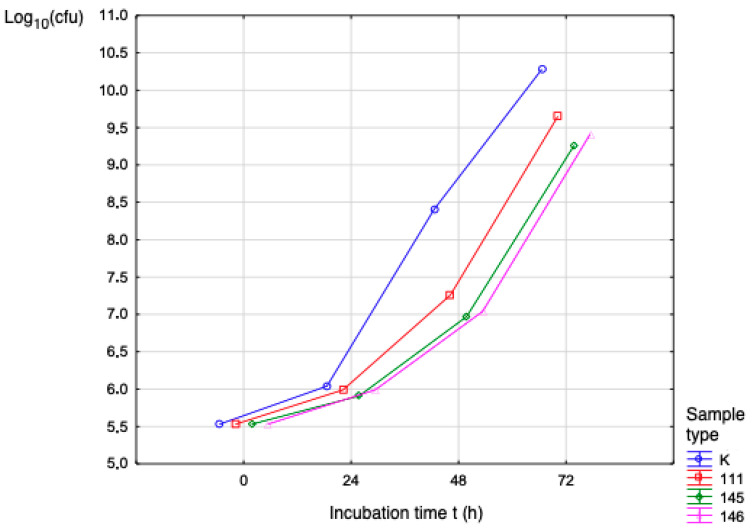
Bacterial activity (*S. aureus*) during the incubation period.

The results of the evaluation of the proliferation of microorganisms in the presence of samples (plates) No. 111, 145, and 146 are presented in Table 9, Table 10 and Table 11. The results were evaluated using bacterial and fungal strains, in the following order: *S. aureus* and *P. aeruginosa* and *C. albicans*. In microbiology, CFUs are defined as colony-forming units. This is a unit used to estimate the number of viable bacterial or fungal cells in a sample. Counting colony-forming units (CFUs) requires culturing the microbes. Only viable cells are counted. The tables show the test samples and the average number of CFUs per incubation period with the calculation of the decadal logarithm, where this is the number of colonies counted on a Petri dish. Figure 9 shows the number of proliferating bacteria (*S. aureus*) per incubation period. From the above graphical results, the average number of CFUs increased in direct proportion to the exposure time. For Sample No. 111, Log_10_(cfu) was 9.65 over the 72 h of incubation time.

**Figure 10:**
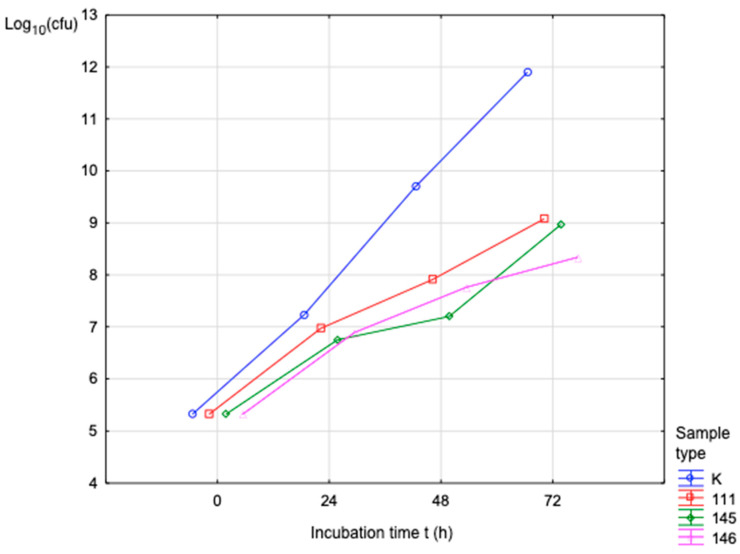
Bacterial activity (*P. aeruginosa*) during the incubation period.

Figure 10 shows that the number of proliferating bacteria (*S. aureus*) over the incubation period increased in direct proportion to the exposure time. For Sample No. 111, Log_10_(cfu) was 9.65 over 72 h of incubation time.

**Figure 11:**
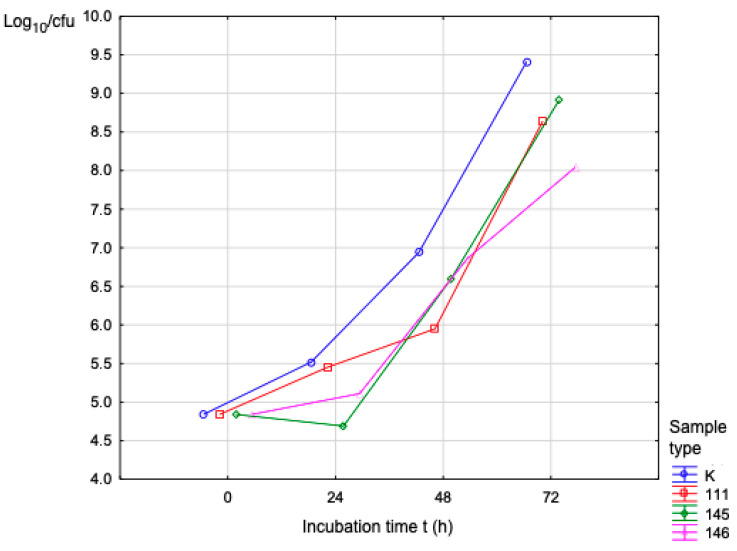
Activity (*C. albicans*) during the incubation period.

CFUs also increased in direct proportion to exposure time when using the fungal strain *C. albicans*. For Sample No. 145, Log_10_(cfu) was 8.91 over 72 h of incubation time. Figure 11 shows the number of proliferating bacteria (*C. albicans*).

### 3.4. Biofilm Testing Analysis

Table 12 gives us an evaluation of the biofilm-forming capacity on the examined sample surfaces (A—smooth side) of *S. aureus*, *P. aeruginosa*, and *C. albicans.*

**Figure 12:**
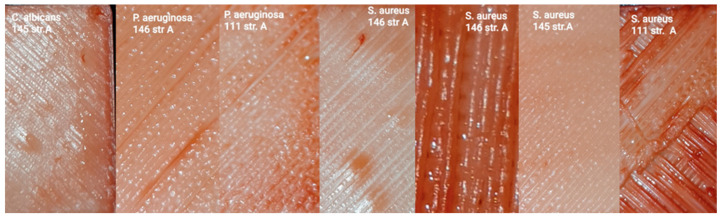
Biofilm formation on the smooth surfaces of the samples.

The formation of biofilm on the examined surfaces of the samples from the smooth side under the action of *C. albicans* and *P. aeruginosa* can be seen in Figure 12. The formation of biofilm on the examined surfaces of the samples from the smooth side under the action of *S. aureus* is also shown in Figure 12.

Table 13 shows the evaluation of the biofilm formation capacity on the examined sample surface (B—rough side) of *S. aureus* and *C. albicans*.

**Figure 13:**
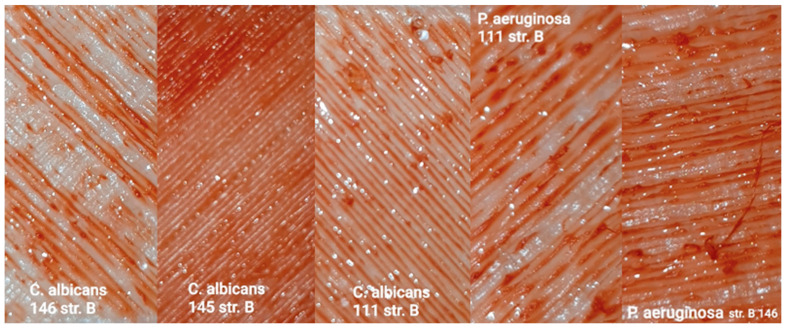
Biofilm formation on the rough sample surfaces.

The biofilm formation on the examined surfaces of the samples from the rough side under the action of *C. albicans* can be seen in Figure 13. The biofilm formation on the examined surfaces of the samples from the rough side under the action of *P. aeruginosa* is also shown in Figure 13. The figures show that the most favorable conditions for biofilm formation are on samples from materials 145 and 111.

### 3.5. Electron Microscopy SEM/BSE Analysis

**Figure 14:**
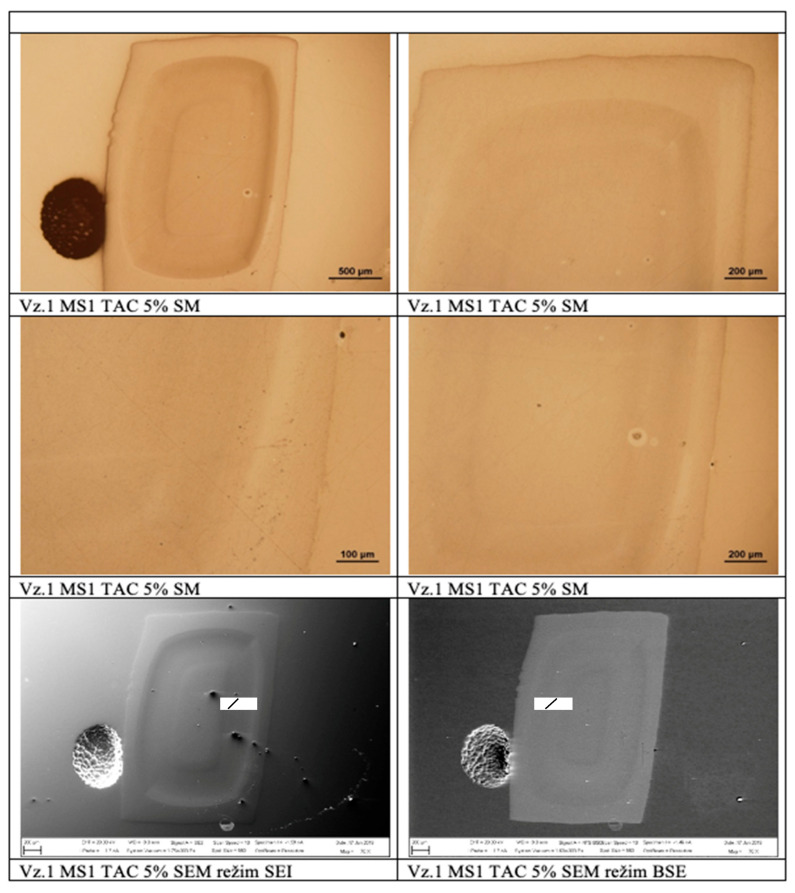
Observation of a sample in SEM and ES modes.

The shape of the pellets for the samples supplied was quadrangular or irregular without filler (Figure 14). A two- to three-layer composite structure (material system combining two or more constituents with different properties to achieve modified or improved behavior) of the polymer matrix was observed for all the samples supplied. No defects in the integrity of the polymer matrix pellet were observed in any of the samples supplied. EDX analysis of the pellets detected a uniform distribution of components throughout the cross-sections of the samples. No sample defects are observed in the images; there is uniform distribution of components.

**Figure 15:**
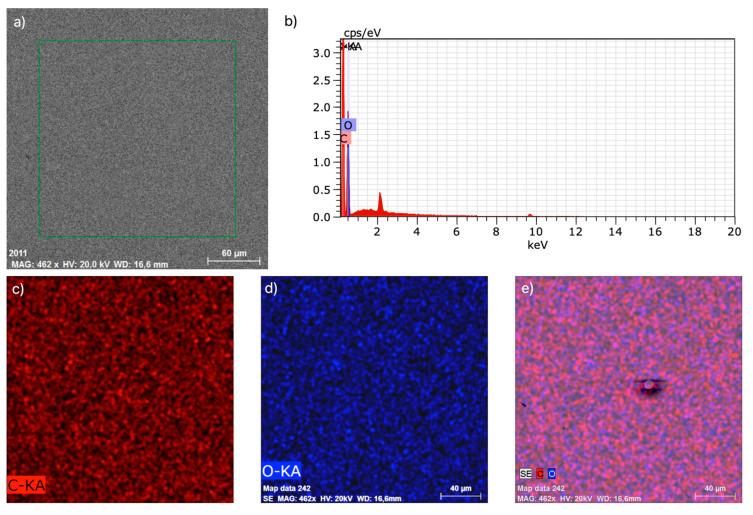
MS1 TAC 5%. EDX area microanalysis: particle center qualitative EDX area microanalysis (**a**), EDX spectrum (**b**), distribution of components (**c**,**d**), qualitative EDX area microanalysis, and composition (**e**).

In Figure 15, we can see the EDX area microanalysis, showing the cut segment for qualitative EDX area microanalysis (a), EDX spectrum (b), distribution of components (c), (d), qualitative EDX area microanalysis, and composition (e). From the above results, we can conclude that there is uniform distribution of components with no signs of defects in the examined samples. MS1 TAC 5% indicates the total atomic concentration of minor elements (5%) detected in measurement spot 1, labeled MS1.

## 4. Discussion

This study stands out for its specificity in terms of the subject matter. Therefore, it is more difficult to discuss the hypotheses and the background from other studies. At the beginning of the research it is necessary to understand what components of the material are involved. In our case, these were biodegradable materials such as PLA/PHB/thermoplastic starch with a plasticizer. At the beginning of the study, it is important to mention the characteristics of the different components of the PLA/PHB mixtures. A study that dealt with the composition and miscibility of the above-mentioned components [45] assumes that PLA is the most used biopolymer in applications in low-pressure parts of the human body. This study addressed several strategies to improve the properties of PLA to expand its applications. Melt mixing approaches are raising considerable interest as they are simple, cost-effective, and readily available. As per our findings, PHB is a good candidate for blending with PLA. The ability of PHB to act as a nucleating agent for PLA improves its mechanical resistance. To enhance the processability of PLA/PHB and achieve more flexible materials, plasticizers are commonly incorporated. Recent approaches to improving PLA–PHB compatibility concentrate on the creation of composites and nanocomposites [46,47,48,49]. Based on this knowledge, the theoretical findings of the above-mentioned studies can be expanded upon and the achieved mechanical as well as biological properties of PLA/PHB components published in this paper can be approached. Looking at another study [50], we can compare and conclude that samples with different concentrations of components were used, which led to similar but not exactly the same results, since the samples contained a mixture of PLA (75 wt%) and PHB (25 wt%) with a polyester plasticizer (Lapol 108) in two different concentrations (5 and 7 wt% per 100 parts of the mixtures). The samples were investigated by TGA, DSC, XRD, SEM, mechanical testing, and biodegradation studies. XRD analysis showed that the original crystal structure of PHB in the PLA75/PHB25 blend was disturbed. In our case, the pattern, i.e., the pellets, should not be disturbed. The DSC curves of PLA and PHB material with a plasticizer showed that the two main components of the mixture were miscible. The values decreased as the amount of plasticizer increased and showed good correlation with the Fox equation. Also, the elongation at break of the PLA/PHB blend was significantly improved by the addition of a plasticizer, which can be confirmed in our conducted research. In a comparable investigation conducted by a different research group [51], a co-rotating twin-screw extruder (Brabender DSE 20) operating at 60 rpm was employed to extrude 1.75 mm ± 0.05 PLA/PHB/nanocellulose (NC) nanocomposite filaments. In contrast, our study utilized a single-screw extruder produced by 3devo under our specific processing parameters, which may influence the filament microstructure, homogeneity, and mechanical performance [52,53,54,55]. We obtained favorable results, and in the comparison we can hypothesize the correctness of using a desktop extrusion system to produce biodegradable filaments with the desired properties. We used slightly different melting temperatures for extrusion, which resulted in better filament flow, as can be demonstrated in the graphs from the preceding chapters of this study. When comparing the applicability of the additive technology, we can conclude that our samples were printed using a TRILAB printer with FDM technology without any signs of defects. We evaluated all hypotheses and comparisons of this study with other studies with high-level articles in the Current Content category. The results are modifiable and the future is open for further possibilities and outcomes regarding this subject, as we can observe from a study where implants were applied to parts in need of bone tissue regeneration [56], where the degradation time is given as a required time of 2 to 6 months. We would like to achieve this time in our further scientific procedures in future implant applications with further tests that would prove this time to be accurate. The research is directed towards the use of another method of non-destructive testing using computer tomography (CT Metrotome). In the future, the objective is to proceed with changing the ratio or adding components for better biological properties and future application in parts of the human body.

## 5. Conclusions

The results achieved will contribute to the understanding of the identified problem in the given area and have greatly contributed to the possibilities for using the given material. The production of medical filaments is a manufacturing process divided into several steps. The first step is to obtain the material in granular form, which is then dried in an oven. After drying, we proceed to the actual production. The optimization of the production is based on the control devices that control the desired properties of the filament, such as the desired thickness of the filament, with a diameter of 1.75 mm. Filament production was carried out on a filament maker from 3devo with the type designation Composer series 450 in a sterile laboratory environment. Three types of filaments were produced with technical designations 111, 145, and 146, where the materials are PLA, PHB, thermoplastic starch, and a plasticizer with different proportions from 25% to 30% of the added plasticizer system. The filament thus produced was suitable for 3D printing of objects using FDM technology, on Trilab’s DeltiQ2 printer. The strength and biodegradation properties of the materials played a major role throughout the research process. The samples were subjected to mechanical testing on equipment from Hegewald & Peschke, where the test speed was set according to EN ISO 527-2:1996 at 2 mm/min. Using an additional extensometer, we recorded the initial measured LO length, the relative elongation, and the percentage elongation. Among the evaluated materials, sample 111 (PLA/PHB with thermoplastic starch and plasticizer) demonstrated the highest mechanical performance, showing a Young’s modulus of 4.63 MPa at 100% infill. Sample 146 displayed a comparable modulus of 4.53 MPa at full infill, while sample 145 exhibited a lower modulus of 1.45 MPa under the same conditions. Microbiological testing consisted of assessing the ability of microorganisms to proliferate in the samples, each with an area of 4 cm^2^ and a thickness of 0.3 cm, using strains of bacteria and fungi: *S. aureus*, *P. aeruginosa*, and *C. albicans*. The biofilm formation test examined the samples at pH = 7, where the samples were dried and subjected to macroscopic examination. The test was carried out in triplicate. Controls were set up to assess the sterility of the test materials and culture medium. The biofilm formation test showed that this undesirable phenomenon had to be addressed by treating the material with chitosan to prevent the formation of a coating on the surfaces of the samples. The CFU data presented in Table 9, Table 10 and Table 11 and Figure 9 already reflect the total viable microbial population, which includes both planktonic and adherent cells. The CFU values allow for an implicit comparison with the initial inoculum levels, effectively representing the relative microbial proliferation and reduction compared to control conditions. In this context, the increase in Log10(CFU) during the incubation period corresponds to the expected microbial proliferation on the surface of the samples, and the observed differences between samples (e.g., sample 111 reached a Log10 of 9.65 after 72 h) indirectly illustrate the dynamics of adhesion and proliferation. No defects in the integrity of the polymer matrix pellets were identified in any of the supplied samples. EDX analysis confirmed a uniform distribution of components across the cross-section, and the SEM images likewise showed no observable structural irregularities, indicating homogeneous material composition.

## Figures and Tables

**Table 1:** Filament production parameters (111) with 25% plasticizer.

Heating Element	4	3	2	1
Extrusion temperature	Temperature range 160–180 °C			
Screw speed	2.0 RPM			
Fan power	80%			

**Table 2:** Filament production parameters (146) with 30% plasticizer.

Heating Element	4	3	2	1
Extrusion temperature	Temperature range 150–170 °C			
Screw speed	4.3 RPM			
Fan power	100%			

**Table 3:** Filament production parameters (145) with 30% plasticizer.

Heating Element	4	3	2	1
Extrusion temperature	Temperature range 150–175 °C			
Screw speed	2.4 RPM			
Fan power	80%			

**Table 4:** Type designations of tested filaments.

Material (Technical Designation)	Plasticizer Content
111	25%
146	30%
145	30% TYPE II plasticizer

**Table 5:** Young’s modulus of elasticity for material 111.

Infill of Material 111	Young’s Modulus of Elasticity (MPa)
100%	4.63
75%	2.84
50%	1.71

**Table 6:** Young’s modulus of elasticity for material 146.

Infill of Material 146	Young’s Modulus of Elasticity (MPa)
100%	4.53
75%	3.8
50%	1.38

**Table 7:** Young’s modulus of elasticity for material 145.

Infill of Material 145	Young’s Modulus of Elasticity (MPa)
100%	1.45 ± 0.17
75%	0.68 ± 0.19
50%	0.47 ± 0.1

**Table 8:** Comparison of materials tested [44].

Material	Young’s Modulus of Elasticity (MPa)
Dental enamel	20–84
Silicone rubber	0.5–5
Material PLA/PHB 111 100%	4.63
Material PLA/PHB 146 100%	4.63
Material PLA/PHB 145 100%	1.75 ± 0.17
Material PLA/PHB 111 75%	2.84
Material PLA/PHB 146 75%	0.04
Material PLA/PHB 145 75%	0.68
Material PLA/PHB 111 50%	1.71
Material PLA/PHB 146 50%	1.38
Material PLA/PHB 145 50%	0.47
Material PLA	3.5
Material PHB	1.8
Tendon	0.8
Cancerous soft tissue	0.02–0.56
Healthy soft tissue	0.0005–0.007

**Table 9:** Evaluation of bacterial activity of the tested materials (*S. aureus*).

Test Material	Incubation Time (h)	Average Number of cfu/mL	Log_10_/cfu
a/Sample No. 111	0	3.4 × 10^5^	5.53
	24	9.9 × 10^5^	5.99
	48	1.8 1 × 10^7^	7.26
	72	4.5 × 10^9^	9.65
b/Sample No. 145	0	3.4 × 10^5^	5.53
	24	8.1 × 10^5^	5.91
	48	9.25 × 10^6^	6.97
	72	1.8 × 10^9^	9.26
c/Sample No. 146	0	3.4 × 10^5^	5.53
	24	9.7 × 10^5^	5.99
	48	1.1 × 10^7^	7.04
	72	2.6 × 10^9^	9.41
Growth control *S. aureus*	0	3.4 × 10^5^	5.53
	24	1.1 × 10^6^	6.04
	48	2.6 × 10^8^	8.41
	72	1.9 × 10^10^	10.28

**Table 10:** Evaluation of bacterial activity of the tested materials (*P. aeruginosa*).

Test Material	Incubation Time (h)	Average Number of cfu/mL	Log_10_/cfu
a/Sample No. 111	0	2.1 × 10^5^	5.32
	24	9.3 × 10^6^	6.97
	48	8.1 × 10^7^	7.91
	72	1.2 × 10^9^	9.08
b/Sample No. 145	0	2.1 × 10^5^	5.32
	24	5.6 × 10^6^	6.75
	48	1.6 × 10^7^	7.2
	72	9.3 × 10^8^	8.97
c/Sample No. 146	0	2.1 × 10^5^	5.32
	24	7.7 × 10^6^	6.89
	48	5.8 × 10^7^	7.76
	72	2.2 × 10^8^	8.34
Growth control *P. aeruginosa*	0	2.1 × 10^5^	5.32
	24	1.7 × 10^7^	7.23
	48	5 × 10^9^	9.7
	72	7.9 × 10^11^	11.9

**Table 11:** Evaluation of bacterial activity of the tested materials (*C. albicans*).

Test Material	Incubation Time (h)	Average Number of cfu/mL	Log_10_/cfu
a/Sample No. 111	0	7 × 10^4^	4.84
	24	2.8 × 10^5^	5.45
	48	9 × 10^5^	5.95
	72	4.4 × 10^8^	8.64
b/Sample No. 145	0	7 × 10^4^	4.84
	24	9.5 × 10^4^	4.69
	48	4 × 10^6^	6.6
	72	8.2 × 10^8^	8.91
c/Sample No. 146	0	7 × 10^4^	4.84
	24	1.3 × 10^5^	5.11
	48	7.2 × 10^6^	6.86
	72	1.1 × 10^8^	8.04
Growth control *C. albicans*	0	7 × 10^4^	4.84
	24	3.3 × 10^5^	5.52
	48	8.8 × 10^6^	6.94
	72	2.6 × 10^9^	9.41

**Table 12:** Evaluation of biofilm formation on side A.

Test Samples	Capacity for Biofilm Formation		
	*S. aureus*	*P. aeruginosa*	*C. albicans*
Sample No. 111	(+++)	(+)	(+)
Sample No. 145	(+)	(+)	(+)
Sample No. 146	(+)	(+)	(+)

Marks: (+)—individual clumps of biofilm formed; (+++)—2/3 of the surface is covered with biofilm.

**Table 13:** Evaluation of biofilm formation on side B.

Test Samples	Capacity for Biofilm Formation		
	*S. aureus*	*P. aeruginosa*	*C. albicans*
Sample No. 111	(+++)	(++)	(++)
Sample No. 145	(+++)	(++++)	(++++)
Sample No. 146	(+++)	(+++)	(+++)

Marks: (++)—1/3 of the surface is covered with biofilm; (+++)—2/3 of the surface is covered with biofilm; (++++)—the whole surface is covered with biofilm.

## Data Availability

The data that support the findings of this study are available from the corresponding author.
